# Upright Posture: A Singular Condition Stabilizing Sensorimotor Coordination

**DOI:** 10.1523/ENEURO.0120-25.2025

**Published:** 2025-07-10

**Authors:** Simon Vandergooten, Laurent Opsomer, Jean-Louis Thonnard, Joseph McIntyre, Philippe Lefèvre

**Affiliations:** ^1^Mathematical Engineering Department, Institute of Information and Communication Technologies, Electronics and Applied Mathematics, Université catholique de Louvain, Louvain-la-Neuve 1348, Belgium; ^2^System and Cognition Division, Institute of Neuroscience, Université catholique de Louvain, Louvain-la-Neuve 1348, Belgium; ^3^Health Unit, TECNALIA, Basque Research and Technology Alliance (BRTA), San Sebastian 20009, Spain; ^4^Ikerbasque, Basque Foundation for Science, Bilbao 48009, Spain

**Keywords:** body tilt, gravity, motor control, targeted arm movements

## Abstract

It has long been hypothesized that the nervous system uses the direction of gravity to align the various sensory systems when interacting with the external world. In line with this hypothesis, systematic drift in hand-path orientation was recently observed during targeted arm motions performed with eyes closed in weightlessness or, on Earth, for longitudinal movements in a supine posture. No such drift was observed in upright posture on Earth. But the precise conditions under which participants exhibit such drift, and the factors that influence the magnitude of the drift, are not yet known. The objective of our study was to investigate if the upright posture, by virtue of being at a biomechanical singularity induced by the force of gravity, represents a unique condition in which drift in hand-path orientation is prevented. Human participants (male and female) performed sequences of repeated point-to-point arm movements between two visual targets aligned with the longitudinal body axis, first with eyes open, then with eyes closed. Participants performed these movements in various body orientations: seated upright, and tilted backward at 45, 90, and 135°. We observed drift in hand-path orientation in the eyes-closed condition when the body was tilted, but not when it was upright. The directions and rates of drift were indistinguishable between the three tilted orientations tested (45, 90, and 135°). These findings support the hypothesis that the upright posture is a unique configuration that facilitates sensorimotor transformations and prevents drift in path orientation when the eyes are closed.

## Significance Statement

Drift in hand paths was investigated during point-to-point arm movements between remembered targets in various body tilts. With eyes closed, drift occurred when the body was tilted with respect to gravity but was prevented when in the upright posture. These observations indicate that the upright bearing is a unique posture in which sensorimotor coordination is the most stable and consistent, supporting recent hypotheses that maintaining the head upright is highly advantageous for stabilizing visuomotor transformations.

## Introduction

Simple arm movements such as reaching to a target require the central nervous system (CNS) to compare the hand and target positions to produce appropriate motor commands. These positions can be acquired via several sensory systems, mainly vision and proprioception, giving rise to redundant estimates that can be optimally combined based on the principle of maximum likelihood estimation (MLE)—which requires the different estimates to be expressed in a common reference frame (Pouget et al., 2002; Sober and Sabes, 2003; Körding and Wolpert, 2004). When visuo-proprioceptive transformations are accurate, the MLE process will reduce noise, without introducing any specific bias. But if they are not, these transformations will introduce bias between the two estimates, and the combined multisensory estimate will shift toward one representation or the other, depending on the relative variance of each signal and the availability of each signal at any given moment. Therefore, one way of limiting errors in the combined estimate is to calibrate the transformations between different sensory systems via a reliable and common marker.

On Earth we can rely on gravity, a constant and ubiquitous signal—widely sensed by the body—that provides a common cue which is thought to facilitate sensorimotor transformations (Paillard, 1971; Tagliabue and McIntyre, 2014; Opsomer et al., 2025). A role of gravity in multisensory integration has indeed been highlighted by previous studies showing that a head-gravity misalignment interferes with eye–hand coordination (Bernard-Espina et al., 2022), verticality perception (Aubert, 1861; Mittelstaedt, 1983; Guerraz et al., 1998), or dynamic balancing (Vimal et al., 2017). Since most daily life activities occur in an upright posture in which the body's axis is aligned with gravity, this natural posture is the basis of any sensorimotor interaction and is thought to elicit a state of enhanced alertness (Paillard, 1971).

In an upright posture, previous studies have shown that participants performing repetitive reaching arm movements toward visual or proprioceptive targets can reliably move the hand in the correct direction when the hand is hidden from view (Brown et al., 2003; Patterson et al., 2017) or even when the eyes are closed (Barden et al., 2005; Opsomer et al., 2025). Not so in weightlessness, where the orientation of the path followed by the hand drifted slowly once the subject closed their eyes (Opsomer et al., 2025). On Earth, despite the presence of gravity, we observed such drift only when both the motion axis and the body orientation were perpendicular to gravity, not when at least one of them was aligned with gravity. To explain those results, we proposed a hypothesis, based on the physical properties of an inverted pendulum, to predict under which conditions the drift in path orientation was expected to occur. We proposed that, when upright, the head is in an unmistakable singular posture where estimation of head orientation is most accurate. Similarly, when moving a handheld object, the vertical axis is a singular axis from which deviations are the easiest to detect. Having the head upright or pushing a weight against gravity would thereby facilitate accurate alignment of proprioceptive signals with respect to the visual surround.

Our hypothesis is based on the premise that the singular characteristics of the upright posture in gravity make it truly unique and unmistakable across sensory modalities, allowing it to be used as a common cue to align reference frames. In our previous study, eliminating gravity removed both distinguishing characteristics (singular head posture and singular movement direction), leading to the appearance of drift in all body tilt and motion axis conditions. The fact that we tested only two body orientations leaves open the possibility, however, for other explanations of the observed data. For instance, drift that varies continuously with the body tilt angle, rather than an all-or-nothing effect of any tilt at all, would also produce significant differences in drift magnitude between upright and supine but would imply an underlying neural mechanism, e.g., tilt-dependent noise or bias, that does not rely on the uniqueness of the upright orientation per se. In the present study, we aimed to answer the question: Does the upright posture provide a truly unique reference to facilitate sensorimotor alignment, or does it represent one endpoint on a continuum of tilt-dependent responses? To this end, participants performed repetitions of targeted arm movements toward remembered target locations placed along their longitudinal body axis in the upright posture and at three different backward tilts of 45, 90, and 135°. We looked for a dose-dependent effect of body tilt on the magnitude and direction of hand-path orientation drift. The results provide further evidence that the CNS relies on the truly singular nature of the upright posture to suppress drift in sensorimotor mappings, while a detailed analysis of the rotation and translation of hand paths provides further insights into what might be the cause of the drift in the first place.

## Material and Methods

### Participants

Twenty-four healthy participants took part in this study. A first cohort of 12 participants (aged 25 ± 6; 6 women; 11 right-handed) participated in the first experiment; a second cohort of 12 (aged 25 ± 2.5; 8 women; 11 right-handed) participated in the second experiment. All participants were naive with respect to the purpose of the study. The experiments were approved by the ethics committee of Université catholique de Louvain, and all participants provided written informed consent before the experiment.

### Task

**Figure 1.**
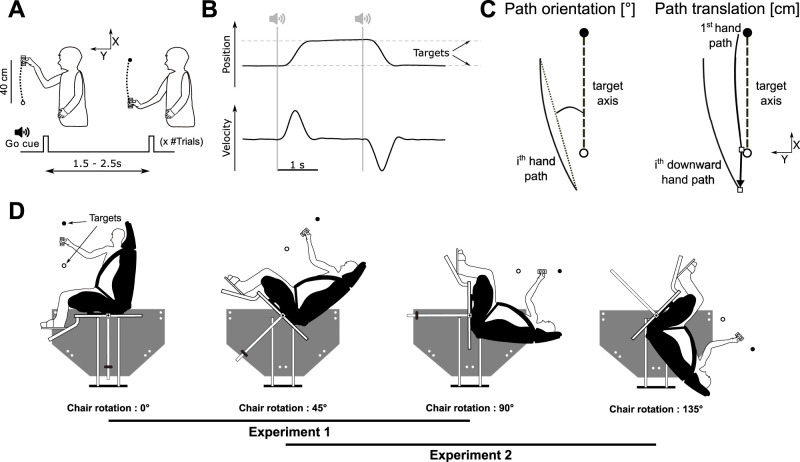
Methods. ***A***, Task consists of point-to-point arm movements toward visual targets with the go signal given by an auditory cue, an egocentric reference frame was used with the *x*-axis parallel to the longitudinal body axis and defined positive toward the head. Participants alternated movements toward the head and feet. ***B***, Exemplar kinematic traces showing the position and velocity along the *x*-axis. ***C***, The hand-path orientation (°) is defined as the angle between the target axis and the line joining the extremities of the hand trajectory. The path translation (cm) is defined as the norm of the 2D vector joining the starting position of the first hand path, which was always next to the lower target, and the endpoint of the i^th^ downward hand path, i.e., when the hand came back to the lower target after a back-and-forth movement. Its components along the *x-* and *y*-axes give the displacement of the hand trajectory along the longitudinal and sagittal body axes, respectively. ***D***, Participants were installed in a chair that could be rotated backward by 45, 90, or 135°. In Experiment 1 (*N* = 12), the body tilts tested were upright, 45 and 90° backward tilts. In Experiment 2 (*N* = 12, different from Experiment 1), the body tilts tested were 45, 90, and 135° backward tilts.

Participants performed point-to-point arm movements with an object held in a precision grip, i.e., between the thumb and index finger (Fig. 1*A*). Movement amplitude (40 cm) was delimited by visual targets positioned on a target mast next to the participant's hand, symmetrically above and below the participant's shoulder and next to the wrist when the arm was fully extended, allowing comfortable movements without complete extension of the arm. Participants moved their hand repeatedly from one target to the other and then back, making a full stop at each target and waiting for the auditory go cue to start each movement. One trial therefore consisted of one movement in one direction from one target to the other, with the ending of one movement being the starting point of the next. Thus, a block of trials consisted of a sequence of movements between two targets, alternating in direction from target 1 to target 2 and from target 2 to target 1. Each experiment was divided into blocks of 30 (Experiment 1) or 24 (Experiment 2) such trials performed in different conditions determined by vision (eyes open or closed) and body tilt (see below), leading to a two-factor within-subjects design. All participants performed the task with their right arm and were instructed to make fast movements, resulting in typical bell-shaped velocity profiles (Fig. 1*B*). In an egocentric reference frame, the movements were always the same with the target mast placed parallel to the longitudinal axis of the body, alternating between movements toward the head and toward the feet. All blocks started with the hand placed next to the lower target, i.e., the target closer to the feet. The delay between go cues was pseudorandomly chosen between 1.5, 1.75, 2, 2.25, and 2.5 s to avoid anticipation, resulting in an average block duration of 60 and 48 s for Experiments 1 and 2. In blocks performed with eyes open (EO), all trials were performed with eyes continuously open; in blocks performed with eyes closed (EC), participants placed their hand next to the starting target, closed their eyes, and then performed all trials with eyes continuously closed.

### Block design

After two to three training blocks performed in upright posture to learn to perform the required movements with eyes open and closed, each participant performed six consecutive blocks in each body tilt, alternating between the two visual conditions (EO and EC) and always starting with the EO condition. In Experiment 1, the participants performed the task at 0° (upright posture), 45°, and 90° of backward body tilt. This permited the comparison of the upright posture, in which the head and motion axes are aligned with gravity, with two tilted conditions in which they are not. Based on initial results that showed drifts almost always in the same direction (toward the feet), we performed a second experiment in which participants performed the task at 45, 90, and 135° of backward body tilt to test whether the direction of the gravitational torque at the shoulder and elbow influences the direction of hand-path orientation drift. In both experiments, the order of body tilts was pseudorandomized and counterbalanced across participants. A 20 s break was imposed between each block, and additional breaks could be requested at any time by the participant. During those breaks, participants could relax their arm. For the 135° body tilt condition, participants were returned to an upright posture after each block performed with eyes closed for 2–3 min for their comfort. Participants were therefore never moved between two consecutive blocks performed with eyes open then closed.

### Equipment

Participants were installed in a custom-built rotating chair consisting of a bucket seat (Evo XL VTR fiberglass seat, Sparco) fixed to a rotating frame. The rotating frame could be set at four different angles: 0, 45, 90, and 135° (Fig. 1*D*). The participants were securely attached to the seat with a 6-point harness (Sparco). The participant's feet were held in place with foot straps. The object held by the subject was a 91 × 37.5 × 48 mm rigid parallelepiped weighting 260 g. In Experiment 1, the position of the hand and object were measured by an infrared marker placed on the nail of the index finger and tracked with a tridimensional (3D) motion tracking system composed of two CODA CX-1 units (CODAMOTION, Charnwood Dynamics). In Experiment 2, four infrared markers embedded in the held object were tracked, and the center of mass of the held object was reconstructed based on the four markers to represent the position of the hand and object.

### Data processing

All analyses were implemented in Matlab R2022A. Position signals were filtered with a dual-pass Butterworth low-pass filter of order 4 with a cutoff frequency of 5 Hz. Signals were expressed in an egocentric reference frame: the *x*-axis was defined positive toward the head and the *y*-axis positive in front of the body. The velocity was obtained by numerical differentiation of the position. Movement onset and offset were defined as the time when the vertical component of the velocity was above or below five percent of movement peak velocity and remained above or below this threshold for at least 50 ms. The analyses presented below focus on the parasagittal plane (*x*–*y* plane) since the contribution of the component along the *z*-axis to the movement amplitude was negligible in all cases (<1%), and we did not find any deterioration of movement characteristics in the absence of visual feedback along the *z*-axis or in the frontal plane.

We computed the hand-path orientation for each trial and hand-path translation for each back-and-forth movement (Fig. 1*C*). Hand-path orientation (°) was defined as the angle between line joining the onset and offset points and the target axis. For the hand-path translation (cm), the start position in the first trial (always at the lower target) was taken as a reference and compared with the following endpoints after executing a back-and-forth movement, i.e., after coming back to the lower target. In other words, the hand-path translation was defined as the norm of the 2D vector connecting the first onset position with the endpoint of the ith downward trial (location_i_ − location_1_, *i* = 1–15 in Experiment 1 and *i* = 1–12 in Experiment 2). This vector was also decomposed into its components along the *x*- and *y*-axis to distinguish the relative displacement of the hand trajectory along the longitudinal and sagittal body axes, respectively. The temporal evolution of those metrics was characterized by a signed drift value (°/s and cm/s), obtained by taking the value of the slope of a linear regression fitted on the temporal evolution of the path orientation and translation for each block separately. The intrasubject variability was computed by taking the standard deviation of the drift for blocks performed in the same condition. To test our hypothesis about the singularity of the upright posture in maintaining consistent hand-path orientation, we also looked at the speed of the drift in path orientation, independent of direction, by computing the unsigned drift rate, i.e., absolute value of the slope of the fitted linear regression. Blocks in the same condition were pooled for each participant after verifying with mixed-effects models that including a random effect of Block to the model did not improve the quality of the fit.

### Statistical analyses

In both experiments, we assessed the effect of body tilt and vision on movement characteristics with a two-way repeated-measures ANOVA with factors Body tilt (three-level factor) and Vision (two-level factor). Mauchly's tests were used to check sphericity assumption and Greenhouse–Geisser corrections were applied when needed. Post hoc analyses were done using paired *t* tests with Bonferroni’s correction. Unpaired Student's and Welch's *t* tests were used to compare the results of Experiment 1 and Experiment 2. Before each test, we verified data normality using Kolmogorov–Smirnov test with Lilliefors adjustment. We assessed the within-individual correlation across conditions using the repeated-measures correlation technique described in Bakdash and Marusich (2017)with the *rmcorr* R package. For all statistical tests, a significance threshold of 0.05 was used. All statistical analyses were performed using RStudio with the *ez* library developed by Michael A. Lawrence. In Experiment 2, one participant's drift in path orientation in the 135° condition was detected as an outlier, i.e., had a drift rate with a *Z*-score greater than three; we therefore removed this participant from the primary analyses, but verified a posteriori that the statistical conclusions did not change when that participant's data were included.

## Results

**Figure 2.**
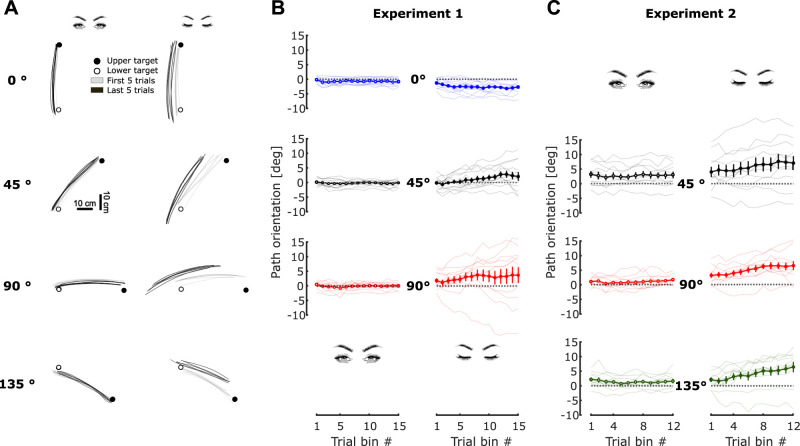
Influence of body tilt and vision on the path orientation of point-to-point arm movements. ***A***, Hand trajectories for illustrative subjects (same participant for 0, 45, and 90° from Experiment 1, another participant for 135° from Experiment 2). Gray and black traces represent the first five and last five trials of the chosen blocks, respectively. Left and right columns are movements performed with and without visual feedback respectively. Each row corresponds to a tilt of the chair, from top to bottom: 0, 45, 90, and 135°. Correspondence of rows and columns with conditions was kept the same for all panels. ***B***, Experiment 1: hand-path orientation (°) pooled across blocks performed in the same vision and body tilt conditions for each participant (*N* = 12; positive, CCW; negative, CW). Trial bins combine two consecutive movements (upward and downward). Thin lines are the individual data, and the thick line is the average across participants. Error bars denote the standard error of the mean (SEM). ***C***, Experiment 2: hand-path orientation (°), same as panel ***B*** (*N* = 11).

While performing the task with visual feedback, participants produced hand-paths that remained precisely aligned with the target axis in all body tilts for both Experiments 1 and 2, as illustrated in the left column of Figure 2*A* with hand trajectories of representative participants. Furthermore, no differences between early (gray traces) and late (black traces) trials were observed. In contrast, in the absence of visual feedback (Fig. 2*A*, right column) body tilt strongly affected the repeatability of the hand-path from one trial to the next. In the upright condition, compared with other body tilts, path orientation was more parallel to the target axis, and stable over time, although the path sometimes gradually translated away from the target axis (Fig. 2*B*, first row). In the tilted postures, i.e., 45, 90, and 135° (Fig. 2*B*,*C*), hand-path orientation drifted substantially away from the target's axis, rotating on average in the counterclockwise direction (when looking at participant's left ear). This rotation was accompanied by a translation toward the feet, i.e., a negative path translation along the *x*-axis (Fig. 4).

**Figure 3.**
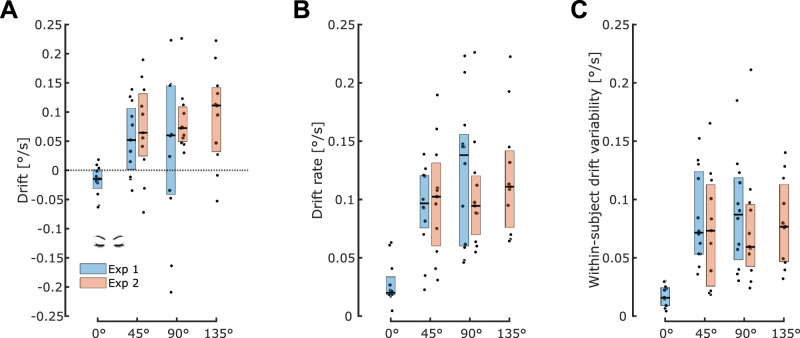
Influence of body tilt on the drift, drift rate, and within-subject drift variability of the path orientation across experiments in the eyes closed condition. ***A***, Comparison of drift value (°/s). Dots represent the average for each individual subject and boxplots display the median and the inter quartile range. ***B***, Same as ***A*** for the drift rate (°/s). ***C***, Same as ***A*** for the within-subject drift variability (°/s).

For Experiment 1, the drift (Fig. 3*A*, blue boxes), the drift rate (Fig. 3*B*, blue boxes), and the within-subject drift variability of the path orientation (Fig. 3*C*, blue boxes) were smaller and closer to zero for the upright posture compared with the two tilted conditions, i.e., 45 and 90° body tilts. To test our hypothesis about the singular nature of the upright posture in preventing the drift in path orientation, we tested if the drift rates were affected by body tilt. Statistical analysis revealed that drift rate was significantly affected by Body tilt $\left(F_{2,22}=18.55,\;\;p<0.001,\;\;\eta_{p}^{2}=0.66\right)$ and Vision $\left(F_{1,11}=82.01,\;\;p<0.001,eta_{p}^{2}=0.83\right)$ with a significant interaction effect between these two factors $\left(\;\mathrm{GGe}\;=\;0.64,\;p[\mathrm{GG}]<0.001,\;\;\eta_{p}^{2}=0.63\right)$. The interaction effect reflects the fact that body tilt affected the drift rate only in the absence of visual feedback $\left(\mathrm{GGe}=0.67,\;p[\mathrm{GG}]<0.001,\;\;\eta_{p}^{2}=0.63\right)$ but not in the presence of visual feedback $\left(F_{2,22}=2.87,\;\;p=0.078,\;\;\eta_{p}^{2}=0.2\right)$. Post hoc analyses revealed that the drift rate was larger at 90 and 45° compared with 0° (*p* < 0.001 in both cases); however, the drift rate at 90° was not significantly different from the one at 45° (*p* = 0.5). The mean drift rates, with eyes closed, were 0.028°/s at 0° (between-subjects 95% CI: [0.016, 0.039]), 0.093°/s at 45° (95% CI: [0.07, 0.11]), and 0.12°/s at 90° (95% CI: [0.08, 0.16]). We verified that the observed drifts were not due to muscular fatigue during the course of a block by comparing the average velocity peaks of the five first and five last movements and found no significant difference in any body tilt conditions with eyes closed (paired *t* tests: 0°: $t_{11}=0.2,\;\;p=0.84,\;\;d=0.02$; 45°: $t_{11}=0.43,\;p=0.67,\;\;d=0.04$; 90°: $t_{11}=0.2,\;p=0.39,\;\;d=0.04$).

For Experiment 2, where only tilted conditions were tested, the drift (Fig. 3*A*, orange boxes), the drift rate (Fig. 3*B*, orange boxes), and the within-subject variability of the path orientation (Fig. 3*C*, orange boxes) were statistically indistinguishable across body tilts (45, 90, and 135° conditions). Statistical analyses indeed revealed a significant main effect of Vision $\left(F_{1,10}=39.55,\;\;p<0.001,\;\eta_{p}^{2}=0.81\right)$ on the drift rate but Body tilt $\left(F_{2,20}=0.40,\;p=0.67,\;\eta_{p}^{2}=0.04\right)$ and the interaction between Body tilt and Vision $\left(F_{2,20}=0.9,\;\;p=0.42,\;\eta_{p}^{2}=0.08\right)$ had no significant impact. We verified that adding the participant whose behavior was detected as outlier in the 135° condition does not change this result. We performed additional testing for differences in drift rate between different body orientations. A single-factor ANOVA showed a main effect of tilt angle (0, 45, 90, 135°), while Scheffé's post hoc testing indicated two homogeneous groups, one containing only the 0° (upright) condition and the other containing all three other tilt conditions, even with alpha = 0.20. While we cannot rule out a dose-dependent effect between the different tilted postures, we can at least claim, based on these homogeneous groups and on visual inspection for Figure 3*B*, that any dose effect is highly nonlinear, with a much larger difference between the 0 and 45° postures than the difference between 45 and 90° or the difference between 90 and 135°. The mean drift rates, with eyes closed, were 0.1°/s at 45° (between-subjects 95% CI: [0.067, 0.134]), 0.105°/s at 90° (95% CI: [0.072, 0.138]), and 0.12°/s at 135° (95% CI: [0.086, 0.154]). These values are comparable with those obtained in Experiment 1 in the shared conditions, i.e., 45 and 90° conditions, and statistical analysis revealed no significant differences in either drift or drift rate between the two experiments (drift: 45°: $t_{21}=-0.56,\;\;p=0.58,\;\;d=0.23$; 90°: $t_{15.185}=-1.23,\;\;p=0.23,\;\;d=0.5$; drift rate: 45°: $t_{21}=-0.4,\;p=0.69,\;\;d=0.17$; 90°: $t_{21}=0.76,\;\;p=0.45,\;\;d=0.32$). Therefore, we reproduced similar results with two different cohorts of subjects. We also verified with paired *t* tests that after grouping cohorts of both experiments, there was still no difference between the 45 and 90° conditions (drift: $t_{22}=0.16$, *p* = 0.87, *d* = 0.03; drift rate: $t_{22}=-1.3$, *p* = 0.2, *d* = 0.35). Similarly to Experiment 1, we also did not find any difference between the velocity peaks of early and late trials in the eyes closed condition (paired *t* tests: 45°: $t_{11}=1.1,\;\;p=0.3,\;\;d=0.06$; 90°: $t_{11}=0.16,\;p=0.88,\;\;d=0.01$; 135°: $t_{11}=0.24,\;\;p=0.82,\;\;d=0.03$).

**Figure 4.**
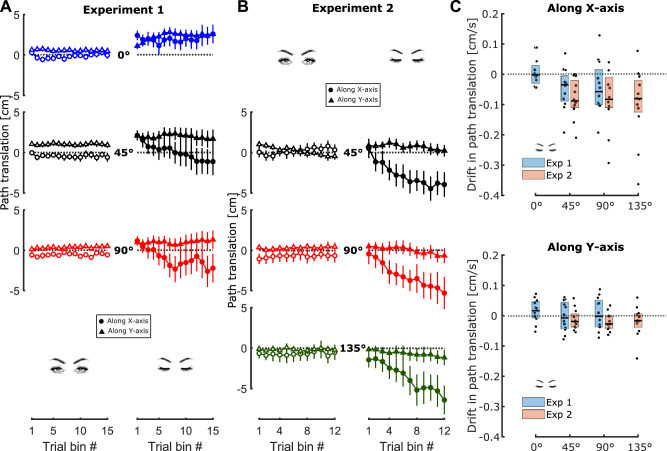
Influence of body tilt and vision on the path translation of point-to-point arm movements. ***A***, Experiment 1: hand-path translation [cm] averaged across participant (*N* = 12) for each body tilt. Left and right columns are the eyes open and closed conditions, respectively. The circle and triangle symbols are the components along the *x*- and *y*-axes, respectively, each row corresponds to a body tilt configuration (from top to bottom: 0, 45, and 90°). Trial bins combine two consecutive movements (upward and downward). Each data point is the average across participants and error bars denote the standard error of the mean (SEM). ***B***, Experiment 2: hand-path translation (cm), same as panel ***B*** (*N* = 12). From top to bottom: 45, 90, and 135° conditions. ***C***, Drift in path translation (cm/s) along the *x*- and *y*-axes in the eyes closed condition. Dots represent the average for each individual subject and boxplots display the median and the inter quartile range.

We also observed substantial translation of the hand paths as measured by the location of the hand at the end of the trajectory close to the lower target. The overall translation of hand paths across trials in the different body tilt and vision conditions is shown in Figure 4. One can observe that the results obtained in the 0° condition (Fig. 4*A*, first row) are different from the ones in the tilted postures (Fig. 4*A*,*B*, second to last rows) in the eyes closed condition (right column of panels *A* and *B*). In the upright posture, the overall translation of the hand-path along the *y*-axis rapidly increased to reach a plateau while no translation of the path occurred along the *x*-axis. The translation of the paths in the upright condition appears to correspond to a jump to a constant bias, rather than a gradual shift in paths over time or repetitions. On the other hand, for the tilted postures, almost no displacement occurred along the *y*-axis, but a significant negative drift was observed for the displacement along the *x*-axis. This corresponds to a progressive shift of the hand position toward the feet. For Experiment 1 (Fig. 4*C*, blue boxes), statistical analysis revealed a marginal effect of Body tilt $\left(F_{2,22}=3.3,\;p=0.056,\;\eta_{p}^{2}=0.23\right)$ on the drift in path translation along the *x*-axis when the eyes were closed while Body tilt $\left(\mathrm{GGe}=0.66,\;p[\mathrm{GG}]=0.41,\;\;\eta_{p}^{2}=0.05\right)$ had no impact on the drift in path translation along the *y*-axis. For Experiment 2 (Fig. 4*C*, orange boxes), statistical analysis revealed no impact of Body tilt for either the drift in path translation along *x*-axis $\left(F_{2,22}=0.19,\;\;p=0.83,\;\eta_{p}^{2}=0.017\right)$ or along the *y*-axis $\left(\mathrm{GGe}=0.6,\;p[\mathrm{GG}]=0.62,\;\;\eta_{p}^{2}=0.03\right)$.

**Figure 5.**
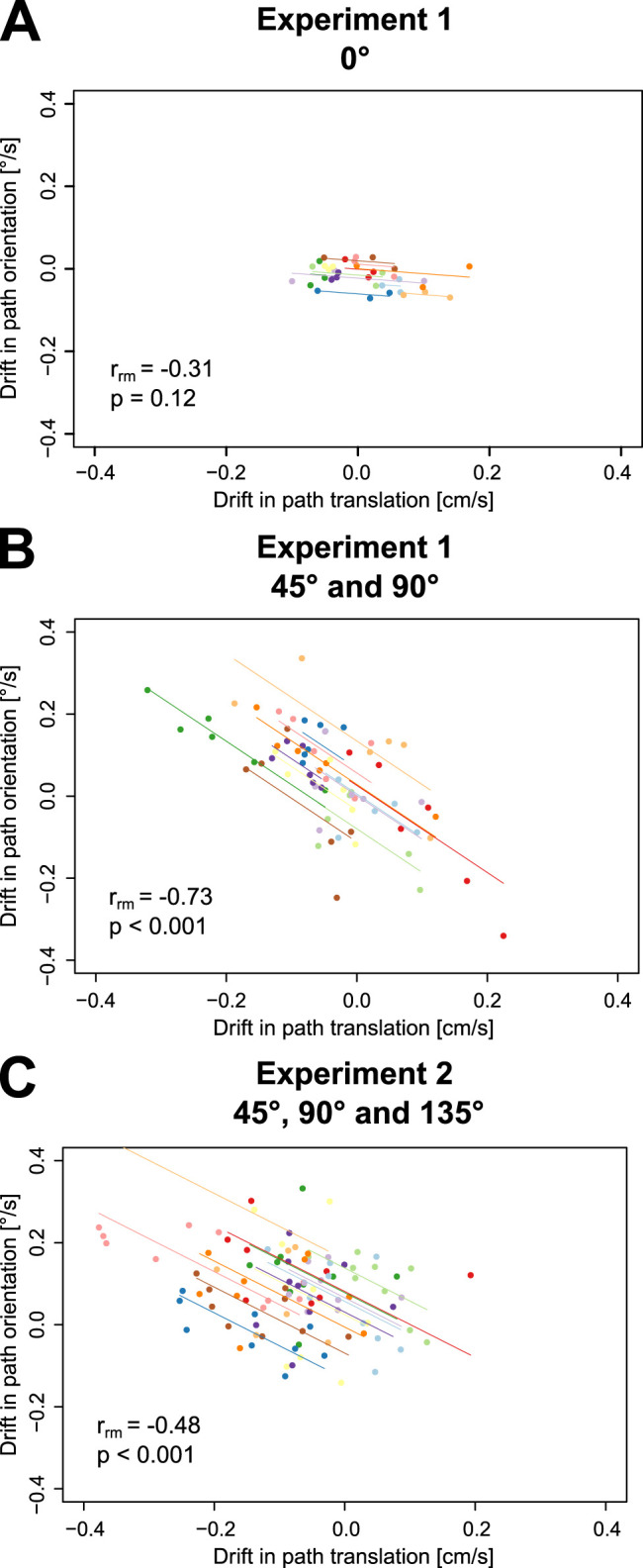
Repeated-measures correlation between the drift in path orientation and the drift in path translation along the *x*-axis in the eyes closed condition. ***A***, Experiment 1 upright (0° condition). Each color is associated with a participant, each data point is the measurement obtained in one block. ***B***, Combined tilted postures from Experiment 1 (45 and 90° conditions). ***C***, Combined tilted postures from Experiment 2 (45, 90, and 135° conditions).

We next looked for a link between the drifts in path orientation and the path translations observed in tilted body orientations by looking for a correlation between these two quantities at the level of each participant and found this to be the case (Fig. 5). In the upright condition (Fig. 5*A*), drifts were not correlated while for both Experiments 1 and 2 with the tilted conditions aggregated, we found strong negative repeated-measures correlation (Bakdash and Marusich, 2017; Fig. 5*B*,*C*). In other words, in the absence of visual feedback in a tilted body orientation, a gradual counterclockwise rotation (while looking at participant's left ear) of the hand trajectory was associated with a translation toward the feet (Fig. 2*A*, second to fourth rows), regardless of whether the body axis was tilted above (45°), below (135°), or aligned with (90°) the horizontal meridian.

## Discussion

In this study, we investigated the uniqueness of the upright posture, compared with tilted body postures, in the ability to maintain consistent hand trajectories during longitudinal reaching arm movements toward remembered visual targets. Our results demonstrated that, in the absence of visual feedback, systematic drift in hand-path orientation occurred in all the tilted body postures tested (45, 90, and 135° backward tilts), with similar magnitude and direction, whereas such drift was absent in the upright posture (“ON/OFF” effect). We also found that the drift in hand-path orientation was associated with a translational drift along the longitudinal axis toward the feet, but only in tilted postures. The presence of drift in all tilted conditions matches a prediction of a recently proposed hypothesis stating that stabilizing the head vertically or pushing directly against gravity provide key cues to prevent the drift (Opsomer et al., 2025). Furthermore, the lack of dependency on body tilt amplitude away from the vertical strengthens the hypothesis that the upright posture is truly a singularity in sensorimotor coordination: the transformations required to convert memorized visual information into motor output are affected whenever the body leaves that posture (Burns and Blohm, 2010; Tagliabue et al., 2013; Bernard-Espina et al., 2022).

The accuracy of arm movements was previously shown to be impaired in various ways in tilted body postures. For example, participants typically undershoot targets during reaching while being tilted forward (Di Cesare et al., 2014) or display endpoint errors in the direction of body tilt when tilted laterally (Tani et al., 2018), but these inaccuracies have not been characterized in terms of a gradual rotation of the sort that is reported here and previously (Opsomer et al., 2025). However, in those studies the visual feedback about the target was typically reset between each movement which prevents observing any accumulation of errors over time, in contrast to what we have seen in our study. In studies where vision was precluded during repetitive arm movements in the upright posture, previous studies did not report drift of hand-path orientation during reaching toward visual (Brown et al., 2003; Patterson et al., 2017) or proprioceptive (Barden et al., 2005) targets but rather a drift in translation. We also observed that in the upright condition the path drifted in translation, rapidly reaching a plateau a few centimeters above and beyond that of the first trial. In all the tilted body postures tested (45, 90, and 135°), however, we observed a more gradual drift along the longitudinal body axis toward the participant's feet, whatever the body tilt. Moreover, the translational drift was shown to be negatively correlated with the drift in path orientation suggesting that both might result from a gradual rotation whose center is not on the hand trajectory itself.

In our original study we postulated that the upright in a gravitational field is truly unique both for the vestibular organs and for the head–neck proprioceptive system, providing a “pop-out” reference that can be used to calibrate transformations between visual and proprioceptive reference frames. We proposed this in contrast to alternative hypotheses in which gravity might directly influence the execution of the motor output. Our experiments rule out the hypothesis that the drifts could be due to improper compensation of the gravitational torques applied to the limb or drift to a more comfortable posture, since rotational drift was outward and translational drift was toward the feet even in a head down posture (i.e., at 135°) or in weightlessness (Opsomer et al., 2025). In addition, we did not find any evidence of muscular fatigue that could explain the drift. On the other hand, the neck muscles could be fully relaxed in all three of our tilted conditions, but not in the upright posture. This could explain the “ON/OFF” phenomenon that we observed. We therefore cannot exclude the possibility that neck proprioception—which is known to affect the precision of upper-limb position sense (Knox and Hodges, 2005; Zabihhosseinian et al., 2015)—could have a more graded influence on upper-limb movement if the head was unsupported when tilted. Most important, however, is the fact that we can exclude an explanation based on tilt-dependent noise in otolithic signals (De Vrijer et al., 2008; Tarnutzer et al., 2009; Vingerhoets et al., 2009), which would otherwise have negated the notion that the upright posture is truly singular with respect to vestibular influences. The hypothesis that the singular upright posture can be used to calibrate sensorimotor transformations remains as a plausible and parsimonious explanation for the drifts (or lack thereof) that we observed here and in our previous studies.

**Figure 6.**
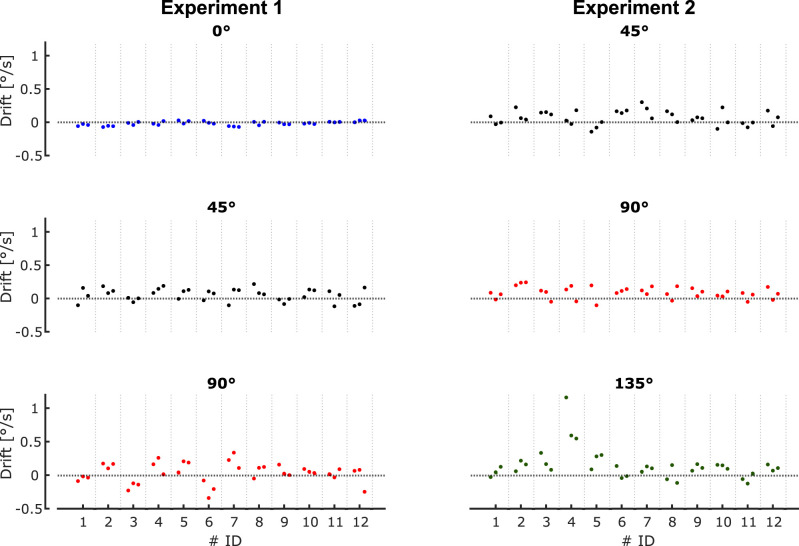
Consistency of drift in path orientation across blocks and body tilts for all participants in the eyes closed condition. Each dot represents the drift of one block, and each participant did three blocks in each body tilt. In Experiment 2, due to a technical issue, Participant 5 in the 90° condition did only two blocks.

Our findings provide new clues to understand the causes of the rotational drift in tilted (and weightless) conditions. It was previously shown that in the supine posture, path orientation drifts as a function of the time elapsed since closing the eyes, not as a function of movement repetition (Opsomer et al., 2025). Therefore, the drift does not stem from motor errors accumulating from trial to trial. A legitimate question one might ask is: could the progressive changes of path orientation over time reflect a gradual misperception of body tilt with respect to the Earth-fixed targets? Indeed, numerous studies have demonstrated that verticality perception is the most accurate with the body upright and is biased in tilted body postures, a phenomenon known as the Aubert–Muller effect (Aubert, 1861; Müller, 1915; Mittelstaedt, 1983). More specifically, participants tend to overestimate body tilt at tilt angles smaller than 60° (E-effect) and underestimate body tilt at larger tilt angles (A-effect), whether the body is tilted in the frontal (Van Beuzekom and Van Gisbergen, 2000) or sagittal (Ebenholtz, 1970; Bortolami et al., 2006) plane. Importantly, this perceptual bias was also shown to drift over time (Tarnutzer et al., 2012, 2013). Thus, one might imagine that drift in path orientation could be the consequence of a drift in the estimation of body orientation with respect to the external world (and thus with respect to the allocentric representation of the targets in memory). This hypothesis predicts, however, different drift directions for head tilts of more than or less than 60° with respect to the vertical, in contrast to what we observed. Despite some intersubject variability, most of the participants showed positive drift on average, similar to what was observed in the supine posture on Earth (Fig. 6) and for both the seated and supine postures in weightlessness (Opsomer et al., 2025).

Conceptually, the brain can plan targeted motions to be performed with eyes closed in a visual reference frame (by comparing a reconstructed visual estimation of hand position to the remembered visual targets) or in a proprioceptive reference frame (by comparing direct proprioceptive information of hand position to the remembered hand position achieved during movements performed with eyes open). It has been proposed that it is advantageous to combine both task representations using optimal multisensory integration principles, even if this requires reconstructing sensory information that is missing in some modalities (Tagliabue and McIntyre, 2011). Under this assumption, the weight given to proprioceptive representations would grow as visual uncertainty increases over time spent with eyes closed. If the visual and proprioceptive reference frames are not aligned, this gradual shift would translate into a drift in hand path. We thus favor a hypothesis wherein drift in path orientation stems from a gradual shift of weighting between visual and proprioceptive reference frames (Smeets et al., 2006), which would no longer be aligned in tilted postures because the saliency of the singular head posture or hand movements disappears. This shift in weighting would be due to the fact that the visual representation of the task degrades in memory over time when the eyes are closed. Therefore, more weight would be progressively given to the proprioceptive information.

But why would visual and proprioceptive reference frames become misaligned in tilted postures, and why would the misalignment be in a particular direction, independent of tilt angle? One possibility is that the memorized proprioceptive target, i.e., the required configuration of the arm to reach the target, is biased toward the most frequent postures. Indeed, gravity constantly pulls downward on our limbs, which could generate a prior for the stable configuration of our arms as being aligned alongside the body. Therefore, in tilted configurations this prior could bias our representation of the target configuration of our arm. Such gravity related bias was already demonstrated for visually remembered postures, i.e., lifted arms were remembered as lower and as closer to the nearest plausible postures (Han et al., 2024). An analogous egocentric prediction of gravity's effect on the limb was also evoked to explain directional asymmetry in the kinematics of arm movements when participant was in a reclined posture with eyes closed (Le Seac’h and McIntyre, 2007). Tilt-independent bias might therefore suggest that the direction of gravity would be predicted as being toward one's feet whatever the tilt angle, leading to a misrepresentation of egocentric hand-path orientation as visual memory fades over time. But such would not be the case in the upright posture, where the singular head posture, combined with salient tactile and proprioceptive cues about the gravitational axis, ensure that proprioception remains aligned with the external world.

In summary, we have demonstrated the unique role of the upright body posture in sensorimotor integration for reaching arm movements along the longitudinal body axis. The lack of tilt “dose dependency” suggests that drift in hand-path orientation and translation, occurring in the absence of visual feedback, could reflect a gradual reweighting between unaligned visual and proprioceptive reference frames whenever the body leaves the upright posture.

## Synthesis

Reviewing Editor: David Franklin, Technische Universitat Munchen Decisions are customarily a result of the Reviewing Editor and the peer reviewers coming together and discussing their recommendations until a consensus is reached. When revisions are invited, a fact-based synthesis statement explaining their decision and outlining what is needed to prepare a revision will be listed below. The following reviewer(s) agreed to reveal their identity: Jérémie Gaveau, Robert van Beers.

Your manuscript has now been reviewed by two expert reviewers. They highlight the report of novel data showing the effect of body orientation on the accuracy and drift of arm movements, which can help to elucidate the role of gravity on motor control. While they both appreciate the contribution to the field, we have some concerns with the current version of the manuscript. In particular, both reviewers bring up some issues regarding other possible interpretations and issues that should be dealt with in a revision. They also highlight that the absence of a significant effect is not a strong support for no differences between two conditions, and that there are more suitable methods. Please refer to the full details of the concerns of the two reviewers which are detailed below as you prepare your revision.

Reviewer 1

This manuscript explores how sensorimotor integration operates during goal-directed arm movements toward memorized visual targets. By manipulating participants' body orientation (via head-feet tilt in the pitch plane), the authors compare movement endpoint kinematics across three postural configurations. Data were collected from two cohorts, each comprising 12 individuals. The findings show that arm movements are most accurate and consistent when participants are seated upright, whereas performance deteriorates in tilted postures. Notably, in the pitched conditions, endpoint directions exhibit a gradual temporal drift. The authors interpret this drift as evidence for dynamic reweighting of visual and proprioceptive inputs, and they argue that their data are incompatible with alternative explanations. While this interpretation aligns with established theories of multisensory recalibration, I find that the results fall short of decisively supporting this particular mechanism over others. The manuscript also refers to the "inverted pendulum" concept of head stabilization in both the abstract and main body of the text, yet it is unclear how this notion is directly tested or validated in the current setup. Overall, the manuscript is clearly written and presents an interesting question, but I have some concerns outlined below.

The discussion of a biomechanical "singularity" related to head stabilization-based on the inverted pendulum model-is intriguing but insufficiently developed. If this idea is central to the explanation, more evidence is needed to link the observed behavioral effects with this specific mechanism. In the current setup, since participants' heads are supported by the chair across all pitch conditions, it is not evident that head instability varies across conditions. Wouldn't this head support mitigate the very instability that the inverted pendulum hypothesis relies on? Furthermore, since head stabilization was not an active component of the task, could the observed drift merely reflect the lack of voluntary head alignment relative to gravity?

I also wonder whether muscular fatigue could contribute to the reported effects. Keeping the arm extended for prolonged periods is physically demanding. Were participants asked about perceived fatigue? The authors suggest that the drift cannot be attributed to fatigue, as it manifests in a constant egocentric direction. This is an important point, but it would be helpful to elaborate on how this observation rules out fatigue as a confounding factor. Alternatively, could the drift reflect a biomechanical adjustment-i.e., participants adopting a progressively more comfortable posture during the block?

Regarding statistical interpretation, the conclusion that no change occurred across blocks appears to rest on a non-significant difference. This is insufficient to claim similarity between blocks. A more appropriate approach would involve equivalence testing to statistically support the absence of a meaningful difference.

Lastly, the manuscript would benefit from a clearer justification for including Experiment 2. What gap or ambiguity from the first experiment does it aim to resolve?

Reviewer 2

This study reports two experiments in which participants were placed in various orientations relative to gravity and had to make sequences of point-to-point arm movements between two targets, with and without vision. It was found that without vision, the location and direction of the arm movements drifted in a rather systematic way over time when the participants were not in an upright orientation. The authors interpret this result as a confirmation of their hypothesis that the upright posture is a unique configuration that facilitates sensorimotor transformations.

The methodology of the study is sound, and the results are very clear and interesting. The manuscript is clearly written, and I especially liked how the authors tried to explain their results in the Discussion. Yet, my main concerns relate to the interpretation of the results. In the Introduction, the author motivate their study by stating that "one way of limiting errors would be to be able to calibrate the different sensory systems via a reliable and common signal allowing the optimal combination of estimates", and this reliable and common signal would be gravity. I have problems following the logic here. In the previous sentence it is mentioned that in order to relate signals from different sensory systems, signals have to go undergo several transformations, each of them adding noise to the reconstructed estimate. I fully agree with that. How and why would such errors be reduced if gravity is used in this process? If all sensory information has to be transformed into a gravity-based reference frame, this requires transformations that add noise. I don't see why using a gravity-based reference frame would lead to smaller errors than using any other reference frame. The authors should clarify their ideas about this.

In the Discussion, the authors state "We thus favor an alternative hypothesis wherein drift in path orientation stems from a gradual shift of weighting between unaligned visual and proprioceptive reference frames as the visual representation of the task degrades in memory (Smeets et al., 2006)." It is not clear to me how this explanation relates to the gravity-based explanation in the previous paragraph. Please clarify.

Another point related to the gravity-based hypothesis is that gravity works only the vertical direction, but we live in a 3D space. This means that if gravity has some beneficial effect on arm movements, this will only act on the vertical components, but it will be ignorant to the two orthogonal horizontal directions. Can gravity also help to improve the horizontal components?

In relation to the previous point, participants had to produce movements in 3D space. Yet, the authors report only results in two dimensions (X and Y). What happened in the Z direction? Were there also errors and drifts in that direction?

An alternative explanation for the results could be that errors in the non-upright postures are made simply because we are not used to make arm movements in those postures. We have a life long experience producing arm movements in upright orientations, so we have learned to make these movements accurately. Or alternatively, our proprioceptive system has learned to be accurate in upright orientations, but not for other orientations. This is all not the case for other postures. Can the authors refute this explanation?

I think that Experiment 2 was conducted to compare the results for the 45 and 135 deg orientations. However, this is never explicitly mentioned. I recommend the authors to mention this, as it will now not for all readers be clear why Experiment 2 was done.
